# The association between authoritarian parenting style and peer interactions among Chinese children aged 3–6: an analysis of heterogeneity effects

**DOI:** 10.3389/fpsyg.2023.1290911

**Published:** 2024-01-08

**Authors:** Dexian Li, Wencan Li, Xingchen Zhu

**Affiliations:** ^1^School of Education, Liaoning Normal University, Dalian, China; ^2^College of Psychology, Liaoning Normal University, Dalian, China

**Keywords:** authoritarian parenting style, peer interactions, social development, gender heterogeneity, age heterogeneity, number heterogeneity

## Abstract

This study explores the effects of authoritarian parenting styles on children’s peer interactions, an aspect often overlooked in the existing literature that primarily focuses on family environmental factors. Data was collected through anonymous child-report questionnaires completed by 2,303 parents and teachers of children aged 3–6 years. The findings reveal that (1) authoritarian parenting significantly hinders children’s peer interactions; (2) the negative effects of authoritarian parenting differ based on gender, age, and family composition: (a) girls generally exhibit higher peer interactions than boys, with authoritarian parenting having a stronger impact on boys’ peer interactions; (b) peer interactions increase significantly with age, and younger children are more susceptible to the negative effects of authoritarian parenting; (c) children with siblings have higher peer interactions, and authoritarian parenting style has a greater influence on their interactions compared to only children. The study discusses potential reasons and provides practical suggestions for families to make informed parenting style choices based on these findings.

## Introduction

Social development plays a crucial role in fostering a well-adjusted personality in children, and the capacity for social support is instrumental in promoting positive psychosocial development, serving as a valuable resource and protective factor. These aspects form the bedrock for establishing positive peer relationships and nurturing physical and mental well-being (Perren et al., 2012; Perren and Diebold, 2017). The quality of peer relationships primarily hinges on children’s proficiency in interacting with their peers, serving as a key indicator of their social development (Zhang et al., 2022). Recognizing the significance of physical and mental development in school-age children, along with the paramount importance of education quality, the new round of preschool education reform in China highlights the Guidelines for the Learning and Development of Children Aged 3–6. These guidelines underscore the importance of attending to children’s peer interactions, as building positive peer relationships constitutes the initial step in children’s social adaptation and plays a pivotal role in enhancing their mental well-being (Tian, 2019).

Existing research both at home and abroad indicates that individuals with an interdependent self-concept often display collectivist traits. They can maintain a collective equilibrium by adjusting their speech and actions to changing situations, fostering harmonious relationships with their peer group (Zhou et al., 2014). Failing to establish appropriate peer interactions can negatively impact children’s social adjustment and interpersonal development in adulthood (Bosacki, 2015). Such adverse peer interactions not only affect children’s social development but also increase the risk of depression and autism (Gong and Liu, 2004). Consequently, scholars have increasingly focused on studying children’s peer interaction abilities, making it a prominent research area in the field of child sociality (Pang et al., 1997). Consequently, considering the widespread presence of authoritarian family structures in China, coupled with the significant focus on children’s peer interactions across various nations, it becomes critically important to investigate the mechanisms through which the authoritarian parenting style influences children’s peer interactions in China, and to examine its diverse effects.

## Literature review

### Parenting styles and children’s peer interactions

The formation and development of children’s peer interactions are complex and multidimensional. Previous scholars, both domestic and international, have explored the relationship between children’s peer interactions and various factors, such as parenting style (Mikami et al., 2010; Ladd and Kochenderfer-Ladd, 2019), teacher-student interactions (Luckner and Pianta, 2011), socio-emotional development (Chen, 2012), and classroom learning (Howe and Mercer, 2007), among other perspectives that encompass multiple domains and dimensions. Parenting style, in particular, plays a fundamental role in children’s social development and significantly impacts peer interactions (Hosokawa and Katsura, 2019; Sahithya et al., 2019; Mak et al., 2020). Parenting styles involve varying attitudes and methods parents use in child-rearing (Darling and Steinberg, 1993). Typically, two central facets define parenting techniques: a parent’s responsiveness and their level of demands (Lamborn et al., 1991; Villarejo et al., 2023). Responsiveness generally refers to a parent’s warmth, active engagement, and support for their child’s individuality (Baumrind, 1991; Climent-Galarza et al., 2022). Conversely, demandingness indicates the degree of rigidity and standards parents establish, aligning with societal or household values (Steinberg et al., 1994; Palacios et al., 2022). Given these two dimensions, researchers have identified distinct parenting categories, such as authoritative, authoritarian, neglectful, and indulgent parenting styles (Lamborn et al., 1991; Steinberg et al., 1994; Villarejo et al., 2023).

Cultural nuances can play a pivotal role in shaping the relationship between parenting techniques and child adaptation (Pinquart and Kauser, 2018; Garcia et al., 2019). Some studies have found that parenting styles in Asian contexts, particularly in Pakistan, tend to lean towards an authoritarian approach. Children raised by authoritative parents exhibit elevated levels of aggressive behavior and emotional instability (Anjum et al., 2019). Within the Chinese cultural and social milieu, a child deemed “well-behaved and aware” is often one who has benefitted from effective parental guidance, encompassing both “discipline” and “instruction.” Rooted in Confucianism, Chinese moral culture emphasizes patriarchal hierarchies and the virtues of righteousness. Parents might resort to stringent disciplinary actions when children do not meet established standards, seeing this firmness as their duty (Chao, 1994). Such exacting parenting helps the young grasp their place in both family and society, aiding in their seamless assimilation into societal norms (Pan and Shang, 2023). Unlike Western parents, Chinese parents often resort to criticism and directives to achieve their expectations of their children when it comes to raising educated, high-achieving family members. This is especially true in areas like academic achievements and the pursuits of the children, showcasing a distinct authoritarian inclination among Chinese parents (Dornbusch et al., 2016; Ren et al., 2022).

Parenting styles encompass a combination of parenting attitudes, methods, behaviors, and emotional expressions conveyed through parents’ responses to their children in daily life, demonstrating cross-situational stability (Darling and Steinberg, 2017). They play a vital role in promoting positive adaptive development (Delvecchio et al., 2020), aligning with Gottman’s research on family emotional life (Gottman et al., 1996). Pivotal research conducted in the United States, primarily focusing on middle-class European-American families, has demonstrated that parenting characterized by a blend of demandingness, as observed in the authoritative style, is correlated with psychosocial benefits (Darling and Steinberg, 1993; Steinberg and Morris, 2001). Conversely, a parenting approach that combines demandingness with a lack of responsiveness, characteristic of the authoritarian style, has been linked to significantly adverse outcomes (Darling and Steinberg, 1993; Steinberg, 2001). Children raised under authoritarian parenting tend to experience social impairments, lack social initiative, have difficulty expressing themselves, struggle to form close friendships, and often face rejection by their peers. Recent research conducted in Europe within middle-class family contexts has also established a correlation between the authoritarian parenting style and adverse consequences (Alcaide et al., 2023; Reyes et al., 2023). As a result, children’s behavior in peer interactions is rooted in the parenting styles they experience.

However, research on ethnic minority groups in the U.S., including African-Americans and Chinese-Americans, reveals a deviation from the European-American family patterns. Authoritarian parenting in these groups is not always harmful and can be beneficial (Baumrind, 1996; Deater-Deckard et al., 1996; Chao, 2001). Martínez et al. (2021) attribute these differences to varied family perceptions and self-esteem. African-American children under authoritarian parenting might feel familial love and connection, unlike their European-American counterparts, who may feel alienated (Baumrind, 1996; Deater-Deckard et al., 1996). This indicates the effects of authoritarian parenting on children’s social behavior vary across cultures. Understanding these parenting styles is crucial for promoting healthy peer interactions and preventing adverse psychological outcomes in children. This study aims to examine the impact of authoritarian parenting on children’s peer interactions in Asian cultures, characterized by collectivism and hierarchical relationships, to provide new insights into scientific parenting.

### Heterogeneous effects of gender, age and number of children

Firstly, as children grow older, their social cognition improves. They exhibit a decrease in the frequency of forceful displays and aggressive conflicts, and gradually realize the benefits of cooperation and sharing, leading to the establishment of intimate relationships (Green et al., 2008). Older children tend to communicate and comprehend more coherently due to better verbal skills, effectively manage socio-emotional conflicts to reduce social barriers, strengthen their social self-confidence, and foster improved peer interactions. However, children raised under authoritarian parenting styles, characterized by ordering, dictating, and controlling, often demonstrate higher dependency and obedience to their parents, lack assertiveness, exhibit poorer group sensitivity, and have lower psycho-theoretical maturity compared to their peers. They tend to view the self and peers as competitors for achievement (Xu et al., 2013), potentially leading to increased aggression. Conversely, children with strong and dominant personalities may find it challenging to maintain long-term peer relationships and experience higher frequencies of peer alienation (Xu et al., 2008).

Secondly, gender differences play a crucial role in child indicators and the manifestation of variations in child socialization (Wei et al., 2011). The peer socialization model suggests that boys and girls exhibit different patterns of social behavior and types of interaction. Girls tend to focus on smaller-scale interactions and are better at providing intimacy, support, and empathy within their social groups, resulting in higher initial levels of pro-social behavior and better peer interactions compared to boys (Van der Graaff et al., 2018). However, boys raised under authoritarian parenting styles have been found to display higher stress responses to group activities and exhibit more negative and hostile emotions. Their withdrawal in peer interactions increases the risk of social impairment and adherence to organizational rules (Wang and Gai, 2020). Another study found that harsh discipline by fathers significantly predicted internal problem behavior and life satisfaction in sons but not in daughters (Wu et al., 2017). This is because boys’ peer groups tend to be larger and engage in rougher play with more physical contact (Wang and Zhuang, 2012).

Thirdly, the one-child family has been the predominant family model in China since the full implementation of the country’s family planning policy in 1979 (Hao, 2012). Only children without siblings have been found to have poorer mental health compared to children with siblings. They face a higher risk of suicidal ideation, self-harm during conflicts, and an increased likelihood of drug dependence (Wang et al., 2019). Due to the absence of other sibling subsystems in the family, parental conflicts and family disputes can directly impact the emotional state of the only child (Hao and Feng, 2002). The adoption of an authoritarian parenting style not only hinders the development of only children but also contributes to negative emotions in their peer interactions, fostering competitive, hostile, and aggressive behavior, leading to a decline in the quality of peer relationships (Tippett and Wolke, 2015). Based on these findings, it is evident that the impact of authoritarian parenting style on children’s peer interactions is influenced by their age, gender, and family structure. This relationship deserves considerable attention in research on family education and children’s social development practices.

### Chinese culture and authoritarian parenting style

Given China’s unique cultural background, the choice of parenting style and the establishment of peer relationships are geographically specific. Chinese parents place greater emphasis on children’s obedience and exert more control over them compared to Western parents (Luo et al., 2013; Ng et al., 2014). Moreover, Asian developing countries, including China, place considerable importance on developing an individual’s ability to inhibit emotions and impulses, viewing overexpression of negative emotions or impulsive behavior as a sign of immaturity (Chen et al., 2005). Children are encouraged to exercise self-control, which is considered a sign of achievement, mastery, and maturity in Confucian philosophy (Ho, 1986).

In conclusion, education in the group home is founded on principles of “Tao” and “Art,” with a primary focus on fostering comprehensive physical and mental development in children. The approach places particular emphasis on promoting emotional stability and appropriate behavior, which leads to the realization of intrinsic and social values in individuals. In summary, numerous studies have explored the theoretical and empirical aspects of authoritarian parenting style and its impact on peer interactions. However, few studies have delved into the profound effects and heterogeneity of authoritarian parenting style on children’s peer interactions during early childhood. To address this gap, the current study aims to investigate the heterogeneous effects of authoritarian parenting style on the peer interactions of 3- to 6-year-old children. The research aims to provide targeted and effective parenting guidance for Chinese family education and raise awareness among families with authoritarian parenting styles about promoting their children’s social development while reducing internalization and externalization problems. Based on the aforementioned context, the following hypotheses have been formulated for this study:

*Hypothesis 1:* Authoritarian parenting style is hypothesized to have a significant negative impact on children’s peer interactions.

*Hypothesis 2:* Gender is hypothesized to moderate the effect of the authoritarian parenting style on children’s peer interactions.

*Hypothesis 3:* Age is hypothesized to moderate the effect of the authoritarian parenting style on children’s peer interactions.

*Hypothesis 4:* Number is hypothesized to moderate the effect of the authoritarian parenting style on children’s peer interactions.

## Materials and methods

### Participants

A total of 2,397 healthy children were recruited from 16 kindergartens, including both public and private ones, located in ten provinces across seven regions in China: North China, East China, South China, Central China, Northwest China, Southwest China, and Northeast China. The recruitment was conducted using stratified and randomized cluster sampling to ensure representative participation in this study. Questionnaires were distributed to parents and teachers of the participating children, with a total of 2,397 questionnaires successfully recovered, resulting in a 100% recovery rate. However, 94 questionnaires exhibited information mismatch and regularity in the answers and were consequently excluded. As a result, 2,303 valid questionnaires were obtained, corresponding to a questionnaire answer validity rate of 96.08%. Among the participants, 1,180 were boys (51.2%) and 1,123 were girls (48.8%). The age range of the children was from 3 to 6 years, and they were divided into two groups: 1,082 children (47%) in the 3–4 years old group and 1,221 children (53%) in the 4 years old or older group. The children were also categorized based on their kindergarten group size, with 784 (34%) in small groups, 782 (34%) in intermediate groups, and 737 (32%) in large groups. Additionally, 6.8% of the children lived in villages (totaling 157), 93% lived in towns (totaling 2,146), and none lived in cities. Furthermore, 9.8% of the children had divorced parents (totaling 98), while 95.7% had intact families (totaling 2,205). In terms of family structure, 46.4% of families had no second child (totaling 1,068), and 53.6% had a second child (totaling 1,235).

### Variables

#### Independent variable

Authoritarian parenting style was assessed in this study using the Parenting Styles & Dimensions Questionnaire-Short Version (PSDQ-Short Version) (Robinson et al., 2001), which was developed by Robinson et al. The PSDQ-Short Version comprises 12 questions related to authoritarian parenting style. These questions gauge parents’ tendencies to display authoritarian behavior and their use of authoritarian practices with their children. This instrument quantifies the extent of authoritarian parenting practices and is completed by all participating parents. For instance, one of the items read, “I would be strict with my child to encourage improvement” (e.g., scolding, criticizing). Respondents rate their agreement on a 5-point Likert scale (1 = never; 5 = always), with higher scores indicating a higher frequency of authoritarian parenting behavior by the parent. The scale has demonstrated robust reliability and excellent psychometric properties when tested with a group of Chinese participants (Li et al., 2012). In this study, the Cronbach’s alpha coefficient for the authoritarian parenting style scale was 0.946.

#### Dependent variable

Peer interactions. In this study, we employed the Peer Interaction Competence Scale for Young Children, developed by Zhang (2002). Several other studies have also utilized this questionnaire (Qin, 2022; Qin and Caizhen, 2022). The scale comprises 24 items that are categorized into four dimensions: social initiative, verbal and non-verbal interactions, social barriers, and pro-social behavior. Teachers completed the questionnaire based on their observations of the children’s daily academic and social performances. One of the sample items reads: “Children can organize a group of their peers to work collaboratively in the classroom.” A 4-point reverse Likert scale was utilized (1 = not at all, 4 = fully), with higher scores reflecting stronger peer interaction competencies. The overall Cronbach’s alpha coefficient for the scale in this study was found to be 0.841, indicating a good level of internal consistency.

#### Control variables

Considering the cultural variations between different regions, we carefully selected individual and family-level control variables that might have an impact on peer interactions and authoritarian parenting style in children aged 3–6 years. At the individual child level, the control variables included gender (coded as boy = 0), age (coded as 3–4 years = 0), class (coded as 1 = junior class), place of residence (coded as rural = 0), and parental marital status (coded as yes = 0 if parents were divorced). At the child’s family level, the control variables encompassed economic conditions, whether the child had a second sibling (coded as no = 0), educational levels of the father and mother, occupations of the father and mother, and the amount of daily time spent with the child by the parents. Table 1 displays the descriptive statistics for all the variables employed in this study.

**Table 1:** Measurement description and descriptive statistical analysis of the variables.

Variables	Description	Sample	M	SD	Min	Max
**Dependent variable**						
Peer interactions	Continuous variable	2,303	2.718	0.407	1	4
**Independent variable**						
Authoritarian parenting style	Continuous variable	2,303	2.925	0.476	1.17	4.83
**Control variables**						
Gender	0 = boy; 1 = girl	2,303	0.488	0.500	0	1
Age	0 = 3–4 years old; 1 = 4–6 years old	2,303	0.530	0.499	0	1
Class	1 = junior class, 2 = middle class, 3 = top class	2,303	1.980	0.813	1	3
Residence	0 = rural; 1 = urban	2,303	0.932	0.252	0	1
Parental marital status	0 = yes; 1 = no	2,303	0.957	0.202	0	1
Family economic conditions	1 = very difficult, 2 = relatively difficult, 3 = moderate, 4 = relatively wealthy, 5 = very wealthy	2,303	3.723	1.051	1	5
Number of children	0 = one child; 1 = more than one child	2,303	0.536	0.499	0	1
Father’s education	1 = below junior high school, 2 = high school/middle school/vocational high school, 3 = college, 4 = bachelor’s degree, 5 = master’s degree and above	2,303	3.529	1.005	1	5
Mother’s education	1 = below junior high school, 2 = high school/middle school/vocational high school, 3 = college, 4 = bachelor’s degree, 5 = master’s degree and above	2,303	3.535	0.972	1	5
Father’s occupation	0 = Managerial (state agencies/institutions) or technical (teachers/engineers/doctors/lawyers); 1 = Other occupations	2,303	0.436	0.496	0	1
Mother’s occupation	0 = Managerial (state agencies/institutions) or technical (teachers/engineers/doctors/lawyers); 1 = Other occupations	2,303	0.449	0.497	0	1
Time spent with the child	1 = 1 h or less, 2 = 1 to 3 h, 3 = 3 to 5 h, 4 = 5 h and above	2,303	3.294	0.875	1	4

### Research process

Prior to commencing the survey, the researchers sought consent from the local education authority and the participating schools. During the survey, essential information about the school campus was gathered from the principal and teachers. The researchers then provided a clear explanation of the study’s objectives and procedures to both parents and teachers before administering the questionnaires. The questionnaires were distributed during parent-teacher meetings conducted in classrooms. Before completing the questionnaires, participants provided written consent and were duly informed about the study’s purpose, confidentiality measures, and the guarantee of anonymity. To ensure consistent and accurate instructions, a trained postgraduate student thoroughly explained the study’s objectives and guidelines to the participants before they filled out the questionnaires. Following completion, the questionnaires were centrally collected and subjected to thorough verification. All research materials used in this study were thoroughly reviewed and approved by the research ethics committee of the corresponding author’s university.

### Research analysis

The data were analysed using SPSS version 26.0 and STATA version 16.0 and consisted of four sections.

Firstly, descriptive statistics and bivariate correlations were performed to assess the relationships between the core variables.

Secondly, in analysing the impact of authoritarian parenting style on children’s peer interactions, the ordinary least squares (OLS) regression analysis was conducted in this process using a stepwise increase in the number of influencing factors, with the model formula:


(1) $$CA_{i}=\beta_{0}+\beta_{1}\rho_{i}+\sum_{k=1}^{k}\beta_{\kappa }x_{i\kappa }+\varepsilon_{i}$$

where $CA_{i}$ represents the score of the ith child’s peer interactions, $\rho_{i}$ represents the score of the ith child’s authoritarian parenting style, $x_{i\kappa }$ represents the kth control variable, $\beta_{0}$ is the intercept term, $\beta_{1}$ is the coefficient of $\rho_{i}$ for the authoritarian parenting style, $\beta_{\kappa }$ is the coefficient of the control variable $x_{i\kappa }$ , and $\varepsilon_{i}$ is the error term.

Thirdly, Generalised propensity score matching (GPSM) model was used to further identify the relationship between authoritarian parenting style and children’s peer interactions. And the robustness of OLS was tested based on the estimation of GPSM with the model equation:


(2) $$Ε\left({ϒ}_{i}\backslash {Τ}_{i},{\hat{R}}_{i}\right)=\alpha_{0}+\alpha_{1}{Τ}_{i}+\alpha_{2}{\hat{R}}_{i}+\alpha_{3}{Τ}_{i}{\hat{R}}_{i}$$

where the treatment variable Τ is authoritarian parenting style and the outcome variable ϒ is peer interactions. Firstly, the conditional probability density distribution of the treatment variable Τ is estimated by GPSM based on the given covariates Χ, and the generalised propensity score R is obtained. Therefore, the two factors that affect both the treatment variable and the outcome variable: the child and the family are selected as covariates. Then, by constructing an OLS regression model with the treatment variable Τ and the generalised propensity score R , the conditional expectation of the outcome variable ϒ was calculated and the coefficients $\alpha_{0}\sim \alpha_{3}$ were obtained. Finally, the range of values of the treatment variable Τ was divided into a number of equally spaced consecutive intervals, and based on the α coefficients obtained in the previous step, the average treatment effect (ATE) of Τ was estimated within each interval (Chen et al., 2014).

Fourthly, the sample data were divided according to gender, age and number of children, and the Fisher permutation test (FP) was used to explore whether the results of the effect of authoritarian parenting styles on peer interaction skills varied according to gender, age and number of children.

## Results

### Common method bias test

All data in this study were collected through self-reports from both parents and teachers, potentially introducing common method bias. To address this concern, Harman’s one-factor test (Zhou and Long, 2004) was conducted. The results indicated the presence of four factors, each with an eigenvalue greater than 1. However, the first common factor accounted for only 21.027% of the variance, which was below the critical threshold of 40%. Consequently, it was concluded that there was no significant common method bias affecting the study.

### The impact of authoritarian parenting styles on peer interactions

Prior to conducting the Ordinary Least Squares (OLS) regression analysis on authoritarian parenting style and peer interactions, a multiple covariance test was conducted on models (1), (2), and (3). Based on the parents’ responses to the questionnaire, the maximum variance inflation factors for models (1), (2), and (3) were 1.00, 2.01, and 5.82, respectively, all of which were below the threshold of 10. These findings indicated the absence of significant multicollinearity issues in models (1), (2), and (3), allowing them to be subjected to OLS regression analysis.

To enhance the scientific validity and reliability of the regression results, multiple adjusted regressions were employed to analyze the impact of authoritarian parenting style on peer interactions by gradually incorporating additional influencing factors (Equation 1). Model (1) only included the core variable of authoritarian parenting style. Model (2) introduced individual child characteristics as a dimension variable in addition to the core variable from model (1). Finally, based on model (2), model (3) included family characteristics as dimension variables (refer to Table 2). The testability index (Table 2) illustrates that as stepwise regression progresses, the models progressively incorporate more factors that affect peer interactions, resulting in an increase in the R^2^ values to 0.325, 0.414, and 0.420, respectively. This indicates that the models are well-constructed, and the inclusion of children’s individual characteristics and family characteristics contributes to a more comprehensive explanation of children’s peer interactions. In each of the models (1), (2), and (3), the estimated coefficients of authoritarian parenting style passed the 1% significance test, and all exhibited negative signs. This suggests that authoritarian parenting style significantly and adversely affects children’s peer interactions. Thus, *Hypothesis 1* is validated.

**Table 2:** Results of the OLS benchmark regression on the effect of authoritarian parenting style on peer interactions.

Variables	(1) Peer interactions	(2) Peer interactions	(3) Peer interactions
Authoritarian parenting style	−0.487^***^ (0.015)	−0.245^***^ (0.020)	−0.225^***^ (0.020)
Gender		0.145^***^ (0.016)	0.143^***^ (0.016)
Age		0.252^***^ (0.018)	0.249^***^ (0.018)
Class		−0.007 (0.008)	−0.008 (0.008)
Residence		0.058^**^ (0.026)	0.055^**^ (0.028)
Parental marital status			0.034 (0.036)
Family economic conditions			0.000 (0.015)
Number of children			0.061^***^ (0.014)
Father’s education			−0.018^**^ (0.009)
Mother’s education			0.017^*^ (0.009)
Father’s occupation			−0.004 (0.031)
Mother’s occupation			−0.005 (0.015)
Time spent with the child			0.009 (0.007)
Sample	2,303	2,303	2,303
*R* ^2^	0.325	0.414	0.420

*p < 0.1, **p < 0.05, ***p < 0.01. The robust standard errors are in parentheses.

### A robustness test of the effect of authoritarian parenting style on peer interactions: an estimation based on the GPSM

Due to the non-normal distribution of the independent variable, authoritarian parenting style from the parent-completed questionnaires, it was standardized within the range of [0,1]. Subsequently, the fractional logit model was employed to estimate the conditional probability density of treatment intensity (Equation 2). The results indicated that the fractional logit model demonstrated a good fit, as evidenced by the Akaike Information Criterion (AIC) value of 0.910. Among the selected control variables, child gender, child age, and whether the family had a second child exhibited the highest regression coefficients for authoritarian parenting style, surpassing other variables significantly. Furthermore, all three variables passed the 1% significance test, signifying their substantial impact on authoritarian parenting style (refer to Table 3).

**Table 3:** Fractional logit regression results for authoritarian parenting style.

Variables	
Gender	−0.325^***^ (0.015)
Age	−0.542^***^ (0.015)
Class	0.111^***^ (0.010)
Residence	0.015 (0.031)
Parental marital status	0.026 (0.037)
Family economic conditions	0.034^*^ (0.019)
Number of children	−0.166^***^ (0.015)
Father’s education	−0.010 (0.011)
Mother’s education	−0.010 (0.011)
Father’s occupation	0.052 (0.037)
Mother’s occupation	0.032^*^ (0.017)
Time spent with the child	0.027^***^ (0.009)
Sample	2,303
AIC	0.910

*p < 0.1, **p < 0.05, ***p < 0.01. The robust standard errors are in parentheses.

**Figure 1:**
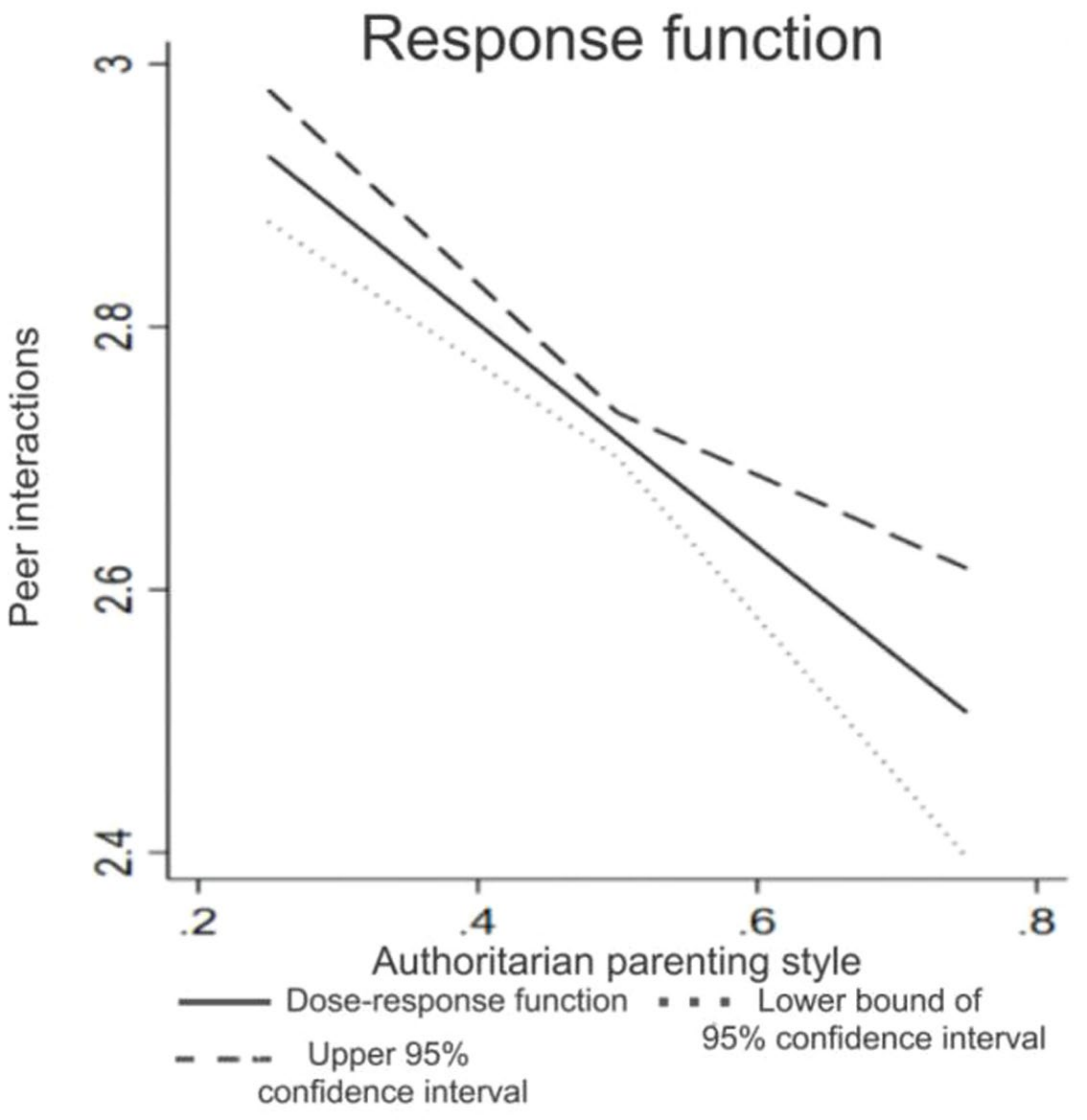
Effects of authoritarian parenting style on peer interactions.

The dose–response plots of authoritarian parenting style (Figure 1) demonstrated a significant decrease in the level of peer interactions as the level of authoritarian parenting style increased, even after controlling for GPSM. This indicates a consistent and robust negative correlation between authoritarian parenting style and peer interactions, which aligns with the findings of the previous OLS regression analysis, reaffirming the strong impact of authoritarian parenting style on peer interactions.

### Heterogeneity in the effects of authoritarian parenting style on peer interactions: based on the gender dimension of children

First, the baseline OLS regression results for model (3) in Table 2 reveal that child gender has a coefficient of 0.143 (*p* < 0.001) in the association between authoritarian parenting style and peer interactions. Secondly, models (1) and (2) in Table 4 display the OLS regression results for the impact of authoritarian parenting style on peer interactions separately for boys and girls. In both cases, the estimated coefficients are statistically significant at the 1% level with negative signs. Notably, the absolute value of the regression coefficient for authoritarian parenting style on boys’ peer interactions (−0.271) is larger than that for girls’ peer interactions (−0.193). Furthermore, the results of Fisher’s exact test for the difference in gender coefficients concerning the effect of authoritarian parenting style on peer interactions are significant at the 5% level. These findings indicate that overall, girls’ peer interactions are higher than those of boys. Moreover, there is a gender heterogeneity effect in the relationship between authoritarian parenting style and peer interactions, with the effect of authoritarian parenting style on boys’ peer interactions being more pronounced compared to girls. As a result, *Hypothesis 2* is confirmed.

**Table 4:** Effects of authoritarian parenting style on peer interactions: gender differences.

Variables	(1) Peer interactions	(2) Peer interactions
	Boys	Girls
Authoritarian parenting style	−0.271^***^ (0.035)	−0.193^***^ (0.023)
Control variables	Yes	Yes
Sample	1,180	1,123
*R* ^2^	0.313	0.245
Fisher empirical *p* value	0.022^**^	

(1) *p < 0.1, **p < 0.05, ***p < 0.01. The robust standard errors are in parentheses. (2) The Fisher empirical *p* value is used to test the significance of the coefficient difference between the groups, which was obtained by 5,000 self-sampling times.

### Heterogeneity in the effects of authoritarian parenting style on peer interactions: based on the age dimension of children

Firstly, the baseline OLS regression results for model (3) in Table 2 reveal a coefficient of 0.249 (*p* < 0.001) for children’s age in the relationship between authoritarian parenting style and peer interactions. Secondly, models (1) and (2) in Table 5 present the OLS regression results for the effect of authoritarian parenting style on peer interactions for two age groups: 3–4 years old and 4–6 years old, respectively. In both cases, the estimated coefficients are statistically significant at the 1% level with negative signs. Notably, the absolute value of the regression coefficient for authoritarian parenting style on peer interactions for children aged 3–4 years (−0.292) is greater than that for children aged 4–6 years (−0.177). Additionally, the empirical *p*-value of Fisher’s exact test for the variability of the age coefficient concerning the effect of authoritarian parenting style on peer interactions is significant at the 1% level. These findings indicate that the level of peer interactions significantly increases with age among children aged 3–6 years. Moreover, there is a child age heterogeneity effect in the relationship between authoritarian parenting style and peer interactions, with the effect of authoritarian parenting style on peer interactions being more pronounced among children aged 3–4 years compared to children aged 4–6 years. Thus, *Hypothesis 3* is supported.

**Table 5:** Effects of authoritarian parenting style on peer interactions: age differences.

Variables	(1) Peer interactions	(2) Peer interactions
	3–4 years old	4–6 years old
Authoritarian parenting style	−0.292^***^ (0.033)	−0.177^***^ (0.025)
Control variables	Yes	Yes
Sample	1,082	1,221
*R* ^2^	0.248	0.132
Fisher empirical *p* value	0.000^***^	

(1) *p < 0.1, ** *p* < 0.05, *** *p* < 0.01. The robust standard errors are in parentheses. (2) The Fisher empirical *p* value is used to test the significance of the coefficient difference between the groups, which was obtained by 5,000 self-sampling times.

### Heterogeneity in the effects of authoritarian parenting style on peer interactions: based on the number dimension of children

Firstly, the baseline OLS regression results for model (3) in Table 2 reveal a coefficient of 0.061 (*p* < 0.001) for the number of children in the family in the relationship between authoritarian parenting style and peer interactions. Secondly, models (1) and (2) in Table 6 present the OLS regression results for the effect of authoritarian parenting style on peer interactions for two groups: children without a second child in the family and children with a second child in the family, respectively. In both cases, the estimated coefficients are statistically significant at the 1% level with negative signs. Notably, the absolute value of the regression coefficient for authoritarian parenting style on peer interactions for children without a second child in the family (−0.300) is larger than that for children with a second child in the family (−0.168). Additionally, the empirical *p*-value of Fisher’s exact test for the variability of the number coefficients concerning the effect of authoritarian parenting style on peer interactions is significant at the 1% level. These findings indicate that children with a second child in the family have significantly more peer interactions than children without a second child in the family. Furthermore, there is a heterogeneous effect of the number of children on the relationship between authoritarian parenting style and peer interactions, with the effect of authoritarian parenting style on peer interactions being more pronounced among children without a second child in the family compared to those with a second child in the family. Thus, *Hypothesis 4* is supported.

**Table 6:** Effects of authoritarian parenting style on peer interactions: number differences.

Variables	(1) Peer interactions	(2) Peer interactions
	Without a second child	With a second child
Authoritarian parenting style	−0.300^***^ (0.032)	−0.168^***^ (0.024)
Control variables	Yes	Yes
Sample	1,068	1,235
*R* ^2^	0.388	0.357
Fisher empirical *p* value	0.000^***^	

(1) * *p* < 0.1, ** *p* < 0.05, *** *p* < 0.01. The robust standard errors are in parentheses. (2) The Fisher empirical *p* value is used to test the significance of the coefficient difference between the groups, which was obtained by 5,000 self-sampling times.

## Discussion

### Negative effects of authoritarian parenting style on peer interactions of 3–6 years old children

The current study has revealed a significant negative predictive effect of authoritarian parenting style on children’s peer interactions, which aligns with family systems theory and is consistent with previous research involving children and early adolescents (Chan et al., 2018; Obimakinde et al., 2019; Zhu et al., 2021). During the child’s interactions with their parents, they observe and imitate the verbal and emotional expressions of family members, and they perceive the reactions of individuals to various stimuli. Consequently, when confronted with relevant situations during group interactions, children draw on the information they have absorbed from their parents to respond in a similar manner, mobilizing their own emotions and executive capabilities. Authoritarian parents’ attitudes are characterized by a lack of respect for their children’s opinions and excessive discipline, with limited feedback, encouragement, and approval. This consistent imposition of unreasonable demands gradually erodes the child’s personality development and reinforces negative parent–child interaction experiences (Liu and Feng, 2023). Over time, this parent–child dynamic can affect the child’s normal responses in peer interactions, rendering them susceptible to intense emotions and aggressive behavior, leading to victimization by peers.

In systems theory, the peer connections of children are seen as a unique system, heavily influenced by family dynamics. The characteristics and behavior patterns within a family, especially those of authoritarian parents, greatly shape children’s interactions with peers (Brown and Bakken, 2011). Children exposed to authoritarian parenting often misinterpret peer actions as hostile, which leads to their alienation and possibly to their withdrawal from social groups (Ros and Graziano, 2018). This social impairment places them at risk of isolation from their peers. Furthermore, in the absence of social support, children might resort to rumination instead of seeking coping mechanisms to handle stress or negative emotions. This can increase their likelihood of developing depressive tendencies (Nolen-Hoeksema, 1991). Authoritarian parenting, characterized by low warmth and high punitive demands, can have detrimental effects on children both at the group and individual levels. High levels of parental authoritarianism correlate with more problematic peer interactions, poor social relationships, and negative social interaction patterns. In structured group settings, some children may react to the restrictive nature of authoritarian parenting by violating established norms or by becoming socially withdrawn and “invisible” to their peers. Both outcomes hinder the development of positive peer interactions, negatively affecting children’s physical and mental well-being and impeding their social development.

### Heterogeneous effects of authoritarian parenting style on children’s peer interactions

This study also revealed heterogeneous effects of gender, age, and the number of children on the negative impacts of authoritarian parenting style on children’s peer interactions.

Firstly, research indicates that girls generally have more peer interactions than boys, influenced by the authoritarian parenting style, which affects boys more significantly. This disparity aligns with Social Role Theory (Eagly, 2013), suggesting societal expectations and gender roles shape emotional and behavioral development. Girls often show obedience, while boys demonstrate assertiveness and defiance, reflecting societal teachings of gender-specific traits (Eagly, 2009). Children internalize these gender characteristics, influencing their focus and processing of gender-related information (Hilt et al., 2010). In peer interactions, girls prioritize relationships and support, using positive strategies during conflicts, thus promoting prosocial behavior (Jahromi et al., 2021). Boys, conversely, focus on game-related emotions, prefer temporary group dynamics, and may resort to aggressive conflict resolution, often exacerbating peer issues. These patterns suggest the need for parental awareness of children’s peer interaction styles, especially among boys, where negative interactions and behaviors are more prevalent.

Secondly, the study’s findings revealed a significant increase in the level of peer interactions among children aged 3–6 years with advancing age. Additionally, the impact of authoritarian parenting on peer interactions was found to be more pronounced among children aged 3–4 years compared to those aged 4–6 years, which aligns with previous research (Zheng, 2018). Physiologically, the prefrontal lobe of a child’s brain undergoes gradual development alongside physical growth. This brain region plays a vital role in emotion regulation and behavioral inhibition, directly influencing children’s emotional responses and controlling their externalized behaviors. The findings of this research align with Gottman’s emotion-centric perspective on parenting approaches and offer insights into discerning if children exhibit positive or negative emotional inclinations (Jespersen et al., 2021). Moreover, the developmental theory of group socialization (Saara, 1951) posits that as children grow older, the proportion of their participation in peer groups significantly increases. Interactions in diverse social situations continually complement and fulfill children’s intrinsic needs for social engagement (Li, 2004). Consequently, their repertoire of peer interaction strategies becomes more varied (Elenbaas, 2019; Ladd and Kochenderfer-Ladd, 2019), and they develop logical thinking when selecting interaction modes that align with the rules of the organization. With reduced dependency on the family for defining their social interactions and values (Sümer et al., 2019), they are more likely to develop self-validation and empathy for others, acquire positive peer interactions, and establish meaningful peer relationships.

Finally, the study’s findings also revealed that children with a second child in the family had significantly higher levels of peer interactions than children without a second child in the family. Moreover, authoritarian parenting style had a stronger impact on peer interactions for children without a second child in the family compared to those with a second child, consistent with a previous study (Cameron et al., 2013). In terms of family structure, children without a second child in the family bear the sole burden of family conflicts and the authoritarian control of their parents (Liu et al., 1988). This unique role may foster individualistic characteristics or an independent self-concept, making them assertive, but also prone to neglecting the feelings and needs of their peers. They may seek to impose their own expectations and achieve personal goals by altering external conditions, exhibiting a strong sense of self and independence. Consequently, in group settings, they may become demanding or even bullies, seeking the initiative and leadership that may be lacking at home and displaying emotional outbursts and problem behaviors that are rejected by their peers. From the perspective of the “erosion-convergence” theory of the dynamics of social influence, the differences in children’s psychological and behavioral outcomes are rooted in variations in family environment and parenting styles. Thus, in light of the recent opening of the three-child policy in China, further comparative investigations and in-depth probes into the effects of different family structures on children’s development are necessary to contribute to the advancement of scientific family and pre-school education.

## Conclusion

After controlling for child- and family-related variables, the results of this study indicate that authoritarian parenting style is negatively correlated with peer interactions. There is gender, age, and number heterogeneity in the negative effects of authoritarian parenting style on children’s peer interactions: (a) Girls’ overall peer interactions are higher than boys’, and authoritarian parenting has a stronger impact on boys’ peer interactions than on girls. (b) Peer interactions among children aged 3 to 6 increase significantly with age, and authoritarian parenting style has a more pronounced effect on peer interactions of 3- to 4-year-olds than on those of 4- to 6-year-olds. (c) Peer interactions are significantly higher for children with a second child in the family than for only children, and authoritarian parenting style has a greater impact on peer interactions for children with a second child in the family than for only children. The implications of these findings are threefold: Firstly, it offers targeted and effective parenting advice for family education. Secondly, this study’s findings contribute to the expansion of family research into a broader international context. By exploring the nuances of family cultures in various countries, we aim to assimilate the most effective aspects of each. Thirdly, the observed diversity in parenting styles across different socioeconomic classes in various countries underscores the need for future research. Such studies could perform in-depth analyses of the relationship between children’s parenting styles, their peer interactions, and socioeconomic class.

## Data availability statement

The raw data supporting the conclusions of this article will be made available by the authors, without undue reservation.

## Ethics statement

The studies involving humans were approved by the Ethics Committee Review Board of Liaoning Normal University. The studies were conducted in accordance with the local legislation and institutional requirements. The participants provided their written informed consent to participate in this study.

## Author contributions

DL: Writing – original draft, Writing – review & editing. WL: Conceptualization, Data curation, Formal analysis, Methodology, Software, Writing – original draft, Writing – review & editing. XZ: Formal analysis, Methodology, Software, Supervision, Writing – original draft, Writing – review & editing.
